# The gut is the epicentre of antibiotic resistance

**DOI:** 10.1186/2047-2994-1-39

**Published:** 2012-11-27

**Authors:** Jean Carlet

**Affiliations:** 1 Réanimation Polyvalente, Fondation Hopital St Joseph, 185 Rue Raymond Losserand, Paris, 75014, France

**Keywords:** Gut, Resistance to antibiotics, SDD, Probiotics, Clostridium difficile, Search, Destroy and restore

## Abstract

The gut contains very large numbers of bacteria. Changes in the composition of the gut flora, due in particular to antibiotics, can happen silently, leading to the selection of highly resistant bacteria and *Candida* species. These resistant organisms may remain for months in the gut of the carrier without causing any symptoms or translocate through the gut epithelium, induce healthcare-associated infections, undergo cross-transmission to other individuals, and cause limited outbreaks. Techniques are available to prevent, detect, and treat the carriage of resistant organisms in the gut. However, evidence on these techniques is scant, the only exception being selective digestive decontamination (SDD), which has been extensively studied in neutropenic and ICU patients. After the destruction of resistant colonizing bacteria, which has been successfully obtained in several studies, the gut could be re-colonized with normal faecal flora or probiotics. Studies are warranted to evaluate this concept.

## 

Resistance to antibiotics will likely be one of the main public health problems of the next decade [1-4]. Gram-negative bacteria, in particular Enterobacteriaceae, have acquired or selected many genes of resistance in the past few years and are now often resistant to third-generation cephalosporins, since they carry extended-spectrum beta-lactamases (ESBLs)[5]. This mechanism of resistance initially emerged in *Klebsiella pneumoniae* but is now even more common in *Escherichia coli*, a micro-organism that lives in the human gut, generally in good intelligence with the host [6]. In several countries, community-acquired infections such as pyelonephritis and peritonitis must be treated with carbapenems, our last line of therapy and a class of drugs heretofore reserved for severe nosocomial infections occurring in the intensive care unit (ICU). Some *K*. *pneumoniae* and *E*. *coli* strains are becoming resistant to carbapenems and require the use of old antibiotics characterized by high toxicity, such as colistin [7]. Resistant Enterobacteriaceae strains are sometimes imported from geographic areas such as Greece, India, North Africa, and Asia [8], in particular after medical tourism. High virulence and resistance may occur in combination, as a lethal duo, as illustrated by the recent outbreak of *E*. *coli* 0104-H4 from contaminated sprouts, which chiefly affected Germany [9]. Hospital strains, such as *Pseudomonas aeruginosa*, *Enterobacter* spp, and *Acinetobacter* spp are highly resistant to ceftazidime, carbapenems, and quinolones [10]. Foodborne nosocomial outbreaks with strains producing SHV and CTX-M-15 have also been described [11]. These data indicate a spiral of increasing resistance that will be very difficult to control [1].

Resistance is an ancient phenomenon related to many factors [12] including the excessive use of antibiotics in both human and veterinary medicine [13,14] and cross-transmission of resistant strains from humans to humans and from animals to humans. A recent study [15] identified ESBL-producing *E*. *coli* strains in up to 80% of retail chicken-meat samples in The Netherlands, a country with very low resistance rates in humans until now. Similar strains have been detected in rectal swabs from humans working with animals [16], and consumers are probably at risk for contamination during meal preparation or consumption of insufficiently cooked meat [17].

The increase in ESBL-secreting *E*. *coli* strains coincides with a marked decrease in the prevalence of methicillin-resistant *Staphylococcus aureus* (MRSA) in several countries [18], indicating that the mechanisms involved differ and that measures instituted to control MRSA are not sufficient to prevent outbreaks of ESBL-secreting organisms. Gram-negative organisms colonize the immense surface area of the human gut. Thus, the amount of susceptible and resistant Enterobacteriaceae in the human body is far greater than the amount of MRSA. Hands can carry high numbers of Gram-negative bacteria [19]. Since compliance with hand disinfection is still very low in many countries [20], cross-transmission can occur easily. In addition, other mechanisms are involved [12,21], such as importation into hospitals of ESBL-secreting *E*. *coli* from the community. In addition, faeces and contaminated sewage from hospitals and livestock can contaminate river water if no waste processing plant is available. This contaminated water may then be used for watering trees or other plants [22,23].

## The gut as the epicentre of bacterial resistance

Knowledge of the gut microbiome, an extraordinarily complex community of organisms, has improved dramatically since the introduction of metagenomics [24]. Many horizontal gene transfers occur among Enterobacteriaceae [25,26] and between pathogens and the gut flora, most notably when the gut barrier is altered [27]. The resistome is also going to be better understood. Under normal conditions, the gut “receives” a large amount of bacteria from the hands, pharyngeal and nasal secretions, water, food, and beverages. Neonates acquire the environmental flora very quickly after birth [28] and in a few cases develop sepsis after translocation of this new flora [29]. In healthy humans, the gut flora in each individual is surprisingly stable [30], and ingested pathogens are cleared fairly easily due to the presence of the commensal flora composed chiefly of anaerobes, most of which are very difficult to isolate. The ingestion of large inoculums of highly pathogenic bacteria (e.g., *Vibrio cholerae*, *Salmonella* spp, or staphylococci) or viruses (e.g., enteroviruses such as echovirus) can destabilise the normal gut microbiome, overwhelm natural defence mechanisms, and induce various clinical symptoms. Antibiotics also very efficiently destabilise the gut microbiome [31-33]. Genetic studies show that even an extremely brief course of antibiotics such as macrolides can induce very long-lived changes in the gut flora, for up to 4 years [34]. Far more will be learned about the gut microbiome in the near future, in particular regarding its stability and alterations.

1) The effect of antibiotics on the digestive flora

Most antibiotics exert a dramatically disruptive effect on the gut microbiome [31-33]. Antibiotics very rapidly kill susceptible bacteria, including *E*. *coli* and the chiefly anaerobic micro-organisms responsible for the barrier function [35]. In parallel, there is an increase in *Candida* and *Pseudomonas* species, as well as in *Enterococci* if a cephalosporin is used [35]. However, depending on the antibiotic, important differences are observed [35]. Antibiotics can select gut micro-organisms initially present in very small amounts, such as *Clostridium difficile*, ESBL-secreting *E*. *coli* (up to 6% of normal individuals are colonised in France [36]), *P*. *aeruginosa*, *Candida* spp, or even *Acinetobacter* spp [37,38]. Collateral damage is worse with antibiotics that have a broad spectrum and/or are largely eliminated via the bile and gut (e.g., ceftriaxone) [39]. In theory, regulatory agencies require an evaluation of the impact of new antibiotics on the gut before granting marketing licences.

2) The gut in critically ill patients

The gut epithelium is very fragile and can be profoundly altered in the most severely ill ICU patients. An increase in gut permeability allows the translocation of micro-organisms into the bloodstream with bacteraemia or candidaemia. Endotoxins and other toxins can also cross the gut barrier. Bacteraemia and endotoxinaemia are among the mechanisms involved in severe sepsis and multiple organ failure [40].

Colonisation of the oro-pharynx, stomach, and distal gut with resistant micro-organisms (enterococci, staphylococci, and Gram-negative bacteria such as *P*. *aeruginosa*) happens very quickly, especially in patients treated with antibiotics. Resistant organisms may be present in very large amounts and are not efficiently cleared from the gut, as transit is often extremely slow. Thus, the gut of ICU patients can be likened to a bacteriological time bomb. The “new” flora (secondary endogenous) [41] can be responsible for nosocomial infections. For instance, gut organisms introduced into the oro-pharyngeal and nasal cavities by gastro-oesophageal reflux may then be inhaled, causing ventilator-associated pneumonia (VAP). This mechanism is the rationale for the use of selective digestive decontamination (SDD) in ICU patients [42].

3) Health-care associated infections with resistant bacteria: where do the bacteria come from?

Resistance to antibiotics can occur in foci of infection during antibiotic therapy (e.g., in the lung in ICU patients treated for VAP) or in the commensal flora, most notably in the gut. Selection of resistant micro-organisms may occur in the gut even after successful treatment of the primary focus of infection [43]. During broad-spectrum antibiotic therapy, the gut may contain high concentrations of most of the resistant Gram-negative and –positive bacteria, including not only Enterobacteriaceae, but also *Pseudomonas*, *Acinetobacter*[37,38], vancomycin-resistant enterococci (VRE), and sometimes even MRSA [44]. Most of the time, these organisms do not result in clinical symptoms and their presence is therefore overlooked. In the most severely ill patients (often in the ICU) and in neutropenic patients, some of the gut micro-organisms (chiefly those present in large amounts) can induce healthcare-associated infections such as pneumonia or translocate through the gut barrier, inducing bacteraemia and/or candidaemia. Life-threatening sepsis may develop as a result in the most severely immunocompromised or neutropenic patients [45].

4) Risk of cross-transmission of gut micro-organisms

The risk of dissemination of multi-resistant Gram-negative bacteria coming from the gut is probably very high, particularly in the ICU [19], since bacteria are found in high concentrations in the rectum. The faeces may contain huge amounts of bacteria (10^8^ per gram of faeces or more), which can then be transmitted via the hands. However, this topic requires more information, since cross-transmission of ESBL-producing *E coli* seems rather limited [46]. Risk factors for dissemination include dependency for toileting, diarrhoea, and living in countries where hygiene is poor. Furthermore, in long-term care facilities, infection control measures are more difficult to implement than in acute care settings.

The very low compliance with hand hygiene rules in most hospitals [47] is a major obstacle to preventing the cross-transmission of resistant and susceptible micro-organisms. Life in hospitals and in the community comprises a succession of small mistakes, which usually have little impact but may increase the risk of cross-transmitting resistant micro-organisms. For example, hand washing after defecation is performed inefficiently or not at all by many individuals. Even in industrialised countries, many people are unaware of the need to decontaminate the hands before and after certain activities. For instance, the hands should be washed thoroughly before touching the clothes and door handles, which requires that a sink be available in the toilet. Simple educational programs designed for the general public are needed. Targeting children may be particularly efficient, as children can then educate the adults in their family [48]. For healthcare professionals in hospitals, alcohol hand rubs or proper use of gloves are very helpful [49].

Contamination and colonisation may also be related to environmental contamination in hospitals, possibly with variations across Gram-negative bacteria [46]. In addition, multi-drug-resistant organisms (MDROs) may be ingested with water or food in the community [23,50,51].

## Are there methods to prevent or treat gut colonisation with resistant gram-negative bacteria?

Although several methods have been discussed, the available information is scant and controversial. Efforts to control bacterial resistance can consist in either prevention or treatment.

1) Oral digestive decontamination for preventing nosocomial infections and antibiotic resistance

SDD has been used chiefly in patients with haematological diseases or neutropenia [52] and in ICU patients, with some measure of success, particularly regarding the incidence of bacteraemia and VAP [53]. The initial goals were to decrease early-onset infections, secondary endogenous colonisation, and the gut endotoxin content.

SDD relies on non-absorbable antibiotics (aminoglycosides, polymyxin E, and amphotericin B) applied to the oro-pharyngeal cavity and administered into the stomach, usually in combination with an intravenous antibiotic (third-generation cephalosporin) for three days. The use of SDD was highly controversial at first, chiefly because early studies found no significant decrease in mortality [53] and many physicians were deeply concerned about the risk of selecting organisms resistant to the drugs used for SDD [54-56],. Recent studies, however, documented a significant decrease in mortality [55,57,58] and a paradoxical decrease in resistance to the antibiotics used locally or systemically [55,58]. Additional studies are clearly needed, since the effect of SDD might be greatly influenced by the level of resistance in a given country [55,58]. It should be noted that the follow up of the studies is relatively short. In summary, SDD has been convincingly proven to decrease the incidence of VAP in the ICU, which is likely to explain the improvement in patient survival. The effect of SDD on antibiotic resistance should be studied in multicentre international studies with a long follow-up. There is now considerable reluctance to use colistin for prevention, as this antibiotic may be the only drug that remains effective to treat some patients with multiple-resistant Gram-negatives.

2) Probiotics

Probiotics have been suggested to maintain or restore gut homeostasis. Probiotics are defined by the World Health Organisation as ‘live microorganisms which when administered in adequate amounts confer a health benefit on the host’. Lactic acid bacteria and bifidobacteria are the most common micro-organism types used as probiotics, although certain yeasts and bacilli may also be helpful. *Saccharomyces boulardii* is a tropical yeast which has been shown to maintain and restore the natural flora in the large and small intestine [59] and is classified as a probiotic. Non-pathogenic *E*. *coli* strains such as Nissle 1917 (EcN) are also classified as probiotics and have been studied in animals, normal volunteers, and elderly patients [60-64].

Studies of S. *boulardii* treatment have generated highly conflicting results. *S*. *boulardii* has been reported to enter the bloodstream in patients with altered gut permeability or marked neutropenia [65]. Studies found that *S*. *boulardii* administration decreased VAP rates [66] and recurrences of *C*. *difficile* infections [67]. A major anti-inflammatory effect in patients with gastrointestinal tract infections or inflammatory bowel diseases was documented in numerous studies.

Many other probiotics have been studied, most notably *Lactobacillus*, alone or in combination with other probiotics. Apart from effects in gastro-intestinal diseases, some studies showed a decrease in VAP, although the results were conflicting [68]. The effects of *E*. *coli* Nissle 1917 (EcN) are similarly unclear. Studies in piglets showed effects on the microbiome and EcN persistence in the gut [60,61]. Persistence of EcN in the faeces was demonstrated in healthy volunteers [62,63]. However, a randomised double-blind study in 69 elderly carriers of quinolone-resistant *E*. *coli* showed no difference with the placebo regarding the persistence of quinolone-resistant strains in the faeces during therapy [64]. Whether probiotics can prevent or treat multi-drug resistant organisms ( MDRO) colonisation remains unknown.

3) Antibiotics with local effects for managing outbreaks

Targeting resistant bacteria with non-absorbable antibiotics is an extremely appealing strategy that has been investigated in patients carrying multi-resistant strains, as well as during outbreaks. Brun-Buisson et al. [69] controlled an ESBL-producing *K*. *pneumoniae* outbreak in the ICU using a combination of colistin and gentamicin. Oro-pharyngeal chlorhexidine baths combined with oral paromomycin (plus an oral antibiotic in patients with urinary tract colonisation/infection) was effective in 76% of patients carrying ESBL-producing *E*. *coli* or *K*. *pneumoniae*[70]. More recently, Perez et al. [71] and Saide-Odes et al. [72] showed in mice and humans, respectively, that polymyxin E plus gentamicin was effective against KPC-producing *K*. *pneumoniae* carriage. Similar efficacy was reported against *Acinetobacter*[73]. Oostdiijk et al. established the efficacy of SDD in eradicating cephalosporin-resistant Enterobacteriaceae from the gut [74]. The data are relatively convincing. However, follow-up is relatively short in all the available studies, and it is difficult to know if recurrences and long term persistence could happen.

4) Faecal microbiome transplantation

Faecal microbiome transplantation consists in administering faecal flora from a normal individual into the gut of a patient with the goal of achieving colonisation with a well-balanced community of organisms. Faecal microbiome transplantation has produced excellent results in patients with *C*. *difficile* relapses [75-77]. In two recent systematic reviews, faecal microbiome transplantation via the oral route or colonoscopy was effective in 83% and 92% of cases of *C*. *difficile* disease, respectively [75,76]. Only 317 patients were treated in all, and the studies were mostly case-series with no control groups. Similar positive results were obtained in young infants with *C*. *difficile* resistant to *S*. *boulardii* and *Lactobacillus rhamnosus*, as well as to many antibiotics [59]. The effect of transplanting *E*. *coli*, instead of the entire normal gut microbiome, has not been widely studied in humans [46-48], and the data are conflicting. Positive results were obtained in animals, particularly mice [78]. Additional studies are needed, both in *C*. *difficile* infections and in other conditions. It would be of great interest to determine whether faecal microbiome transplantation prevents MDRO re-colonisation after decolonisation.

5) Beta-lactamase treatment

Oral treatment with enteric-coated beta-lactamases has been used in dogs and in humans [79,80] in an attempt to prevent the appearance of antibiotic resistance in the gut during intravenous ampicillin administration. The desired effect is destruction by the beta-lactamase of residual antibiotic in the distal gut, to prevent the acquisition of resistance without affecting systemic drug levels. Both studies were encouraging, in particular the study in human patients, which included a control group [79,80]. However, the development of this compound has been stopped due to a lack of resources. This topic deserves further attention, since the concept is appealing.

6) The search, destroy, and restore concept

The ‘search and destroy’ concept was used initially in The Netherlands to prevent and treat MRSA colonisation and infection. Patients at risk for MRSA carriage were screened, and cultures of the pharynx and sometimes of the skin were performed [81]. The patients were isolated until the results became negative. Patients with MRSA colonisation were treated with nasal mupirocin, often combined with chlorhexidine baths. This strategy may be among the reasons explaining the very low prevalence of MRSA in The Netherlands [64]. The search and destroy strategy has also produced favourable outcomes in Ireland, Denmark, and other Scandinavian countries [82,83]. Similar strategies are now used more widely not only for MRSA, but also for resistant Gram-negative bacteria producing ESBL or carbapenemases [69-74]. At-risk patients (coming from high-prevalence countries or having had numerous hospital stays) are screened then isolated until the results are available. No official guidelines have been issued for treating colonised patients. Decolonisation was achieved in a few studies [69-74].

It would seem logical to add a third step after the search and destroy steps, consisting in gut re-colonisation with normal flora or *E*. *coli*. This search, destroy, and restore strategy would be expected to decrease the risk of re-colonisation with MDROs [84]. Studies of this strategy are warranted. Several methods could be evaluated, including the administration of probiotics and faecal microbiome transplantation.

## Conclusion

The gut plays a prominent role in the development of antibiotic resistance, allowing the hidden selection and multiplication of resistant micro-organisms in the community, long-term care facilities, and hospitals [84]. The emergence of resistant micro-organisms in the gut may be related to the ingestion of highly pathogenic micro-organisms or to antibiotic-induced alterations in the gut microbiome [85]. The resistant organisms then contaminate the environment via the faeces [86]. Cross-transmission of the resistant strains can occur relatively easily if strong hygiene measures are not taken. However, these measures are not easy to implement [87].

Methods for preventing or controlling the appearance of antibiotic resistance in the gut include SDD, local antibiotic therapy, probiotics, and elimination of residual antibiotics from the gut (using enzymes or adsorbents). Studies are warranted to determine whether combining several of these methods in a search, destroy and restore strategy is effective and safe. The available data on the gut as a reservoir for antibiotic resistance are uncomplete, and much more will have to be learned before recommendations are made.

## Competing interests

Dr Jean Carlet is a consultant for da Voltera, Biomérieux, Thermofisher, Astra-Zeneca, Astellas, and Aromatechnologies.
